# Editorial: Investigating Grammar in Autism Spectrum Disorders

**DOI:** 10.3389/fpsyg.2018.01004

**Published:** 2018-06-19

**Authors:** Stephanie Durrleman, Anna Gavarró

**Affiliations:** ^1^Département de Psycholinguistique Université de Genève, Geneva, Switzerland; ^2^Departament de Filologia Catalana Autonomous University of Barcelona, Barcelona, Spain

**Keywords:** autism spectrum disorders, syntax, finitiness, DLI, c-command, pragmatics, Wh- movement, control

Autism Spectrum Disorder (ASD hereafter) is a neurodevelopmental condition characterized by deficits in communicative and social skills. The vast majority of research on language in ASD has focused on pragmatic difficulties, while less is known about structural aspects of language in this population. Work on syntax and phonology is not only sparse, but the heterogeneity in these grammatical domains has moreover led to conflicting reports that they are either intact or impaired. More remains to be understood about variations in grammatical profiles in ASD, as well as the relation of grammar to other cognitive abilities.

The purpose of this Frontiers Research Topic is to bring together investigations of grammar in ASD suggesting novel meaningful ways to parse the associated heterogeneity. Topics addressed include experimental investigations of domains delayed in Developmental Language Impairment (DLI), comparisons of the grammatical profiles of ASD with those of other language-impaired populations, careful analyses of subgroups, and the grammar-cognition interface.

Regarding domains delayed in DLI, the paper by Modyanova et al. focuses on the production of tense marking in a large study with Language-Impaired (ALI) and Language-Normal (ALN) English-speaking children with ASD. As a general finding, ASD children show no problem with subject-verb agreement or case, indicating that impairment does not affect syntax in a broad sense. The authors conclude that, while the ALN are not different from their verbal- and non-verbal-matched controls, the ALI are indeed impaired in tense production, even more severely than in the DLI population for which tense marking is well-established as a marker of impairment.

In this same vein, Sukenik and Friedmann investigate movement to non-argumental positions in ASD and DLI by means of subject and object relative clause elicitation, reading and rephrasing object relatives, and sentence repetition. While the results for the two populations appear similar, under closer scrutiny the errors of ASD and DLI participants are different in nature, with a distinct error pattern, syntactically driven in DLI but not in ASD, and consistently arising in individuals with DLI, but not in ASD. This result challenges the claims for a common source of language deficits in the two pathologies.

Khetrapal and Thornton examine linguistic competence in ASD via experiments tapping into knowledge of the structural relation of c-command. Previous work had suggested that children on the spectrum exhibited difficulties with this structural constraint, given that they struggled with reflexives. The high-functioning children in the current study, however, performed on a par to typically developing (TD) peers in computing both operator scope (negation and disjunction) and binding (reflexives), allowing the authors to conclude that the hierarchical relation of c-command is intact in high-functioning children with ASD.

Some contributions revisit the issue of pragmatic difficulty alongside structural language in ASD. Andrés-Roqueta and Katsos explain that the seemingly contradictory reports on pragmatic competence in ASD make sense once we separate linguistic pragmatics from social pragmatics. Linguistic pragmatics would be required by certain tasks assessing informativeness, metaphors, and idioms, and affected to the extent that structural language and vocabulary are impaired. Social pragmatics involves the ability to take others' perspectives into account and is affected to the extent that there is also a Theory of Mind (ToM) deficit.

Similarly, the paper by Janke and Perovic studies the interpretation of sentences with control in three conditions: complement control and temporal adjunct control, both syntactic dependencies, alongside controlled verbal gerund subjects, a pragmatic dependency. The two groups tested, high-functioning ASD children and a TD control group, performed in the same way in complement control and controlled verbal gerund subjects, and only marginally differently in temporal adjunct control, showing that syntax is unimpaired and pragmatics is not pervasively impaired.

Jyotishi et al. turn to wh-questions and argue that the lag in comprehension of these structures in ASD seems in part grammatical and in part social-pragmatic. It appears partially grammatical because in their study (i) it is observable despite a procedure itself reducing social/pragmatic demands and (ii) improved performance on wh-questions is predicted by higher performance on SVO word order. At the same time, social-pragmatic scores also play a role in predicting both ASD and TD groups' later comprehension of wh-questions.

Peristeri et al. investigate narrative production in high-functioning children with ASD to reveal that higher linguistic abilities of some groups with ASD boost their narrative skills, both in syntactic and pragmatic domains. However, persistent difficulties in certain pragmatic domains can be observed alongside good language skills, suggesting that the latter do not necessarily allow children with ASD to overcome their pragmatic challenges.

In an attempt to elucidate the linguistic heterogeneity in ASD, Wittke et al. explore different language subtypes in a large group of 5-year-old children with ASD. Going beyond standardized tests for defining language groups, the authors also probe grammatical problems via a detailed analysis of natural language samples. The findings suggest the presence of several linguistic subtypes, ranging from intact language to minimally verbal. The identification of children showing high non-verbal reasoning and vocabulary in the presence of low grammatical abilities provides support for a specific impairment in grammar in this ASD subgroup.

Burnel et al. address the language/cognition interface by assessing belief attribution in neurotypical adults and adults with Asperger Syndrome (AS). In their results, neither neurotypicals nor those with AS were significantly affected by verbal shadowing; however adults with AS performed more slowly than neurotypicals, and were more disrupted in ToM tasks when asked to repeat complement clauses than relative clauses. The findings suggest that ToM reasoning in adults with AS involves compensation because of persistent ToM difficulties, a compensation which may specifically solicit complementation syntax.

The body of research gathered here increases our understanding of the grammatical strengths and weaknesses in ASD. The contributions carefully elucidate the relations between grammar and other areas of cognition, as well as unveil the similarities and differences of grammar in ASD compared to other conditions. The result is a volume that provides new ways to think about language and communication in ASD, and beyond, which should be of interest to both linguists and clinicians.

## Author contributions

All authors listed have made a substantial, direct and intellectual contribution to the work, and approved it for publication.

### Conflict of interest statement

The authors declare that the research was conducted in the absence of any commercial or financial relationships that could be construed as a potential conflict of interest.

